# Evaluation of short and mid-term clinical outcomes in patients with aortic coarctation treated with self-expandable stents

**DOI:** 10.1038/s41598-024-62607-w

**Published:** 2024-05-23

**Authors:** Mahmoud Mohammadzadeh Shabestari, Ali Eshraghi, Farnaz Hakim Attar, Fereshteh Ghaderi, Hoorak Poorzand, Amir Hossein Mohammadzadeh Shabestari, Behzad Alizadeh, Negar Morovatdar, Bahram Shahri, Hedieh Alimi, Mohammad Tayyebi, Arash Gholoobi, Vahid Reza Askari, Yousef Ali Garivani, Mohammad Mohammadzadeh Shabestari, Vafa Baradaran Rahimi

**Affiliations:** 1 Department of Cardiovascular Diseases, Faculty of Medicine, Mashhad University of Medical Sciences, Mashhad, Iran; 2 Islamic Azad University, Central Tehran Branch, Tehran, Iran; 3 Vascular and Endovascular Surgery Research Center, Mashhad University of Medical Sciences, Mashhad, Iran; 4 Division of Congenital and Pediatric Cardiology, Department of Pediatrics, Faculty of Medicine, Mashhad University of Medical Sciences, Mashhad, Iran; 5 Clinical Research Development Unit, Imam Reza Hospital, Mashhad University of Medical Sciences, Mashhad, Iran; 6 Pharmacological Research Center of Medicinal Plants, Mashhad University of Medical Sciences, Mashhad, Iran

**Keywords:** Self-expandable stent, Aortic coarctation, Uncovered stent, Sinus-XL, Peak gradient, Cardiology, Interventional cardiology

## Abstract

The present study aimed to evaluate the outcomes of percutaneous treatment of aortic coarctation using self-expandable uncovered Nitinol stents. We conducted a retrospective clinical data review of all patients with aortic coarctation and treated with self-expandable uncovered Nitinol stents at our institution between 2009 and 2019. The gradient pressure across the coarctation site was measured using aortography. Follow-up echocardiography and computed tomography angiography were performed to assess possible stent complications. A total of 127 stents were successfully implanted in 125 patients (64.8% males) with a mean age of 35.36 ± 11.9 years. The gradient across the coarctation site decreased significantly from 67.48 ± 14.79 to 5.04 ± 3.01 mmHg (P < 0.001) after self-expandable stent implantation. Systolic blood pressure (SBP) decreased significantly from 175.53 ± 15.99 to 147.22 ± 12.83 mmHg (P < 0.001) after self-expandable stenting. There were no major technical or clinical complications, including balloon rupture, aneurysmal formation, infection, secondary stent migration, thrombosis, death during the procedure, and in-hospital mortality. On a mean follow-up of 48 ± 23.6 months (12–120 months), the gradient [from 59.43 ± 15.42 to 3.72 ± 1.38 mmHg (P < 0.001)] and SBP [from 175.53 ± 15.99 to 127.99 ± 7.82 mmHg (P < 0.001)] decreased significantly. There was no mortality, aneurysmal formation in the stent site, dislocation, or aortic re-stenosis requiring intervention during mid-term follow-up. Treatment of aortic coarctation using a self-expandable uncovered nitinol stent is safe and effective with promising mid-term outcomes.

## Introduction

Coarctation of the aorta is considered one of the most common congenital heart defects, accounting for 5 to 8% of all cardiovascular malformations^1^. It is accompanied by several anomalies, including a bicuspid aortic valve, ventricular septal defect, mitral valve prolapse, and Lazarus syndrome^2^. The prevalence of aortic coarctation is two times higher in men than in women. It manifested with clinical symptoms, such as cardiogenic shock, heart failure in childhood, headache, lameness, and systemic hypertension in early youth and adulthood. The diagnosis of the aortic coarctation is performed by clinical symptoms and imaging^3,4^. There are two indications for therapeutic intervention for aortic coarctation^5^. First, if the diameter of the stenosis was less than or equal to 50% of the diameter of the aorta in the diaphragmatic area, regardless of the calculated gradient. Second, in case of the difference in the peak pressure gradient at the site of stenosis is more than 20 mm Hg even in the presence of normal blood pressure^6,7^.

Treatment of aortic coarctation is performed by balloon angioplasty, stenting, or surgery. The surgery method for treating aortic coarctation in early adults has less than 1% mortality and a 9% risk of postoperative complications, such as suture aneurysms and later restenosis^8,9^. Additionally, surgery may cause transient or permanent neurological damage in older adults initiated by degenerative changes in the aorta. Surgery also has a negative mental and emotional impact on patients, the need for ICU admission, and a longer length of hospitalization^10,11^. Nowadays, stent implantation has been approved as an alternative method to surgery. Multiple advantages have been emphasized for balloon-expandable stents, including radial support to the aortic wall, lower risk of relapse, aortic septum dissection, and aneurysm formation. However, balloon-expandable stents have complications, namely stent fracture and dislocation^12,13^.

Currently, self-expandable nitinol stents are gaining attention in the management of aortic coarctation. Furthermore, some pieces of evidence showed mitigated aortic wall complications as well as promising effects during follow-ups^14^. However, the long-term efficacy and safety of these stents are still not well-studied. In this regard, the present study aimed to evaluate the short-to-mid-term results of patients with aortic coarctation treated with self-expandable nitinol stents.

## Methods

### Ethics

Research ethics approval was obtained from Mashhad University of Medical Sciences (approval code. IR.MUMS.MEDICAL.REC.1399.583). Written informed consent was obtained from the adult participant or the child’s parents or legal guardian and signed by them all. In addition, the study was carried out in accordance with the Declaration of Helsinki.

### Conceptualization of the study

We evaluated all patients with coarctation of descending thoracic aorta treated with self-expandable stenting between 2009 and 2019 in Razavi and Imam Reza hospitals affiliated with Mashhad University of Medical Sciences.

### Inclusion and exclusion criteria

We included patients with ages more than ten years, weight more than 35 kg, pressure gradients exceeding 20 mmHg through the coarctation site, and resistant hypertension or symptomatic hypertension at a young age^15,16^. The exclusion criteria were difficulty accessing the lesion site due to the structural and technical conditions of the lesion, severe and simultaneous hypoplasia of the descending and transverse aorta, and aortic atresia in the coarctation site.

### Sinus-XL stent characteristics

The Sinus-XL stent, a self-expanding uncovered nitinol stent (OptiMed), is designed with a closed cell, adequate flexibility, easy placement, and maximal radial force to manage coarctation. There are different sizes available, from 16 to 34 mm in diameter to 30–100 mm in length. In order to deliver, the Sinus-XL stent needs a 10 F sheath and 0.035-inch guidewire. Interestingly, radiopaque markers are embedded in the inner sheath of the stent, which facilitates the placement. Additionally, it has an anti-jump specification in order to maintain stability and prevent sudden jumps^17,18^. The indications criteria to use the stent were having more than 50% narrowing of the aortic lumen distal to the left subclavian artery at imaging or having hypertension and pressure gradients exceeding 20 mmHg through the coarctation site. In addition, no specific contraindication has been reported for using this device, and its use is highly recommended in all patients.

### Procedural intervention

Interventional procedures were carried out in a standard catheterization or hybrid operating room. Before the procedure, patients were given 325 mg of aspirin and a mild intravenous sedative. After that, all patients underwent retrograde femoral artery catheterization. Following local anesthesia, a 6F sheath was placed into the femoral artery. Then, a 0.035-inch hydrophilic guidewire with a multipurpose (A1) catheter was passed retrograde through the femoral route along the aorta through the coarctation. After fixing the A1 catheter, the hydrophilic guidewire was exchanged with a stiff guidewire. In order for aortography in the aortic root and thoracic aorta, the A1 catheter was exchanged with a pigtail catheter, and an aortogram was performed in the left anterior oblique or caudal view.

In the next step, a 6 F femoral sheath was exchanged with a 10 F femoral sheath along the stiff guidewire. Then, the stent was advanced along the stiff guidewire following an intravenous bolus injection of 5000 IU of heparin. Stenting with the usual sizes of 18 to 32, depending on the size of the aorta at the coarctation site and proximal to the stenosis, was performed optimally. Then, the stent was opened at the appropriate site with intermittent contrast injections. Finally, based on the initial information obtained from the anatomy of the stenting site, a non to semi-compliant balloon was used for post-dilation based on the nominal size of the balloon with simultaneous fluoroscopy. No additional proceeding was performed in patients with residual gradient < 10 mmHg, except aortography and final fluoroscopy^19–21^.

After the procedure, the patients were monitored for at least 24 h in the CCU department. Furthermore, aspirin (80 mg/day) was prescribed for the first 6 months after stent implantation.

### Evaluation of outcome

The demographic characteristics, antihypertensive drugs, and systolic blood pressure (SBP) of patients were recorded. The gradient pressure across the coarctation site was measured using aortography. All patients were followed up at 1 week, 3, 6, and 12 months, and then annually to determine blood pressure and antihypertensive drugs. All patients enrolled in this study had at least 12 months of follow-up. In addition, the gradient pressure after self-expandable stent implantation was evaluated using Trans-thoracic echocardiography (TTE) or Trans-esophageal echocardiography (TEE). Computed tomography angiography (CTA) was performed during mid-term follow-up to evaluate possible complications after stenting.

### Statistical analysis

Data were analyzed using the SPSS version.22 statistical software (SPSS Inc., Chicago, Illinois) and Graph Pad Prism 8.01 software (Graph Pad Software Inc., USA). Data were shown as means ± SD or numbers with percentages according to the nature of parametric and non-parametric, respectively. The comparison between continuous variables was performed using paired t-tests for parametric data. The levels of P values (P) ≤ 0.05 are regarded as statistically significant.

## Results

This study evaluated 150 patients with Coarctation of the descending thoracic aorta treated with self-expandable stenting. However, 25 patients did not participate in the follow-ups and were excluded. Finally, we investigated the results of 125 patients during the 48 ± 23.6 months (12–120 months) follow-up.

### Demographic characteristics

The demographic and clinical information of the studied patients is summarized in Table 1. The mean age of studied patients was 35.36 ± 11.9 (ranging from 15 to 59); 10 (8%) patients were under 18 years old, and 115 (92%) patients were ≥ 18 years old. Furthermore, 64.8% of patients were male, and the complaint of all 125 studied patients was uncontrolled hypertension despite the use of antihypertensive drugs (Table 1).

**Table 1:** Demographic characteristics of studied patients.

Characteristic (N = 125)	Mean ± SD or N (%)
Age	
Years	35.36 ± 11.9 (15–59)
< 18	10 (8%)
≥ 18	115 (92%)
Gender	
Male	81 (64.8%)
Female	44 (35.2%)
Hypertension	125 (100%)
Antihypertensive therapy	
Total	125 (100%)
Mono-therapy	9 (7.2%)
Multi-therapy	116 (92.8%)
Kidney disease	0 (0%)
Hypoplasia	12 (9.6%)
Discrete	111 (88.8%)
Re-operation	2 (1.6%)
Re-stenting	0 (0%)

### Perioperative and immediate results

**Figure 1:**
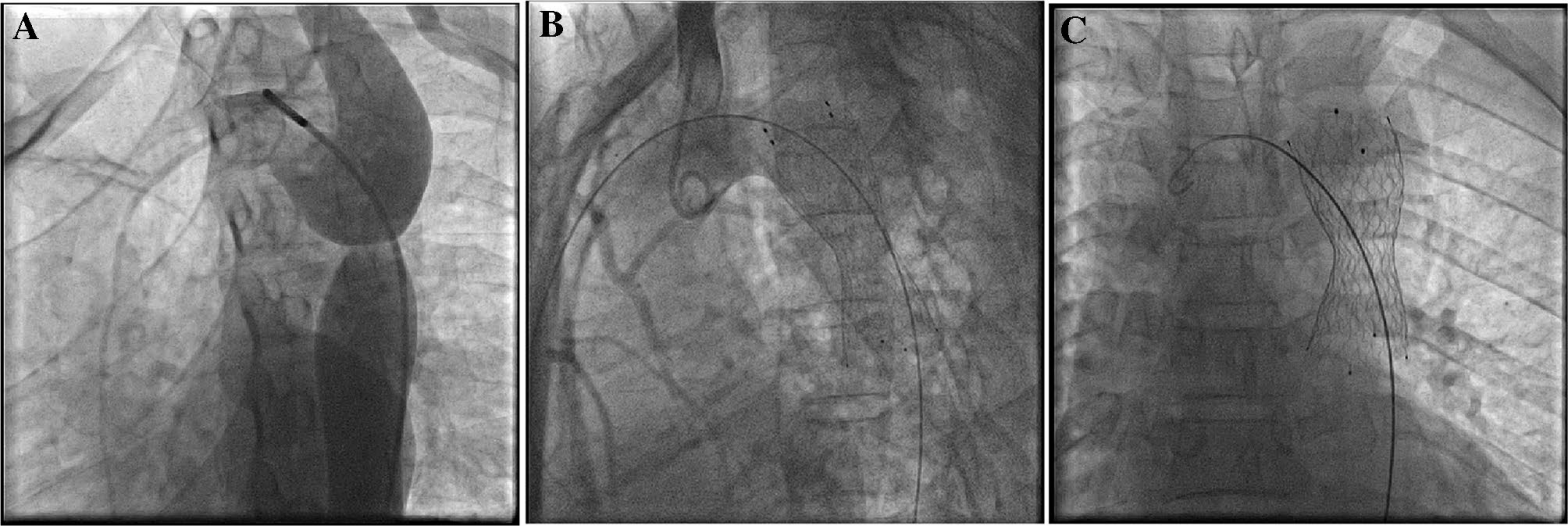
Intraoperative angiographic monitoring; (**A**) before and (**B,C**) after stent implantation.

Our results showed successful operation was achieved in all patients (125, 100%) following self-expandable stenting implantation, and 127 stents were used (Fig. 1). The stent diameter was between 18 and 32 mm, and the stent length was 60 mm, which was fixed for all patients.

**Figure 2:**
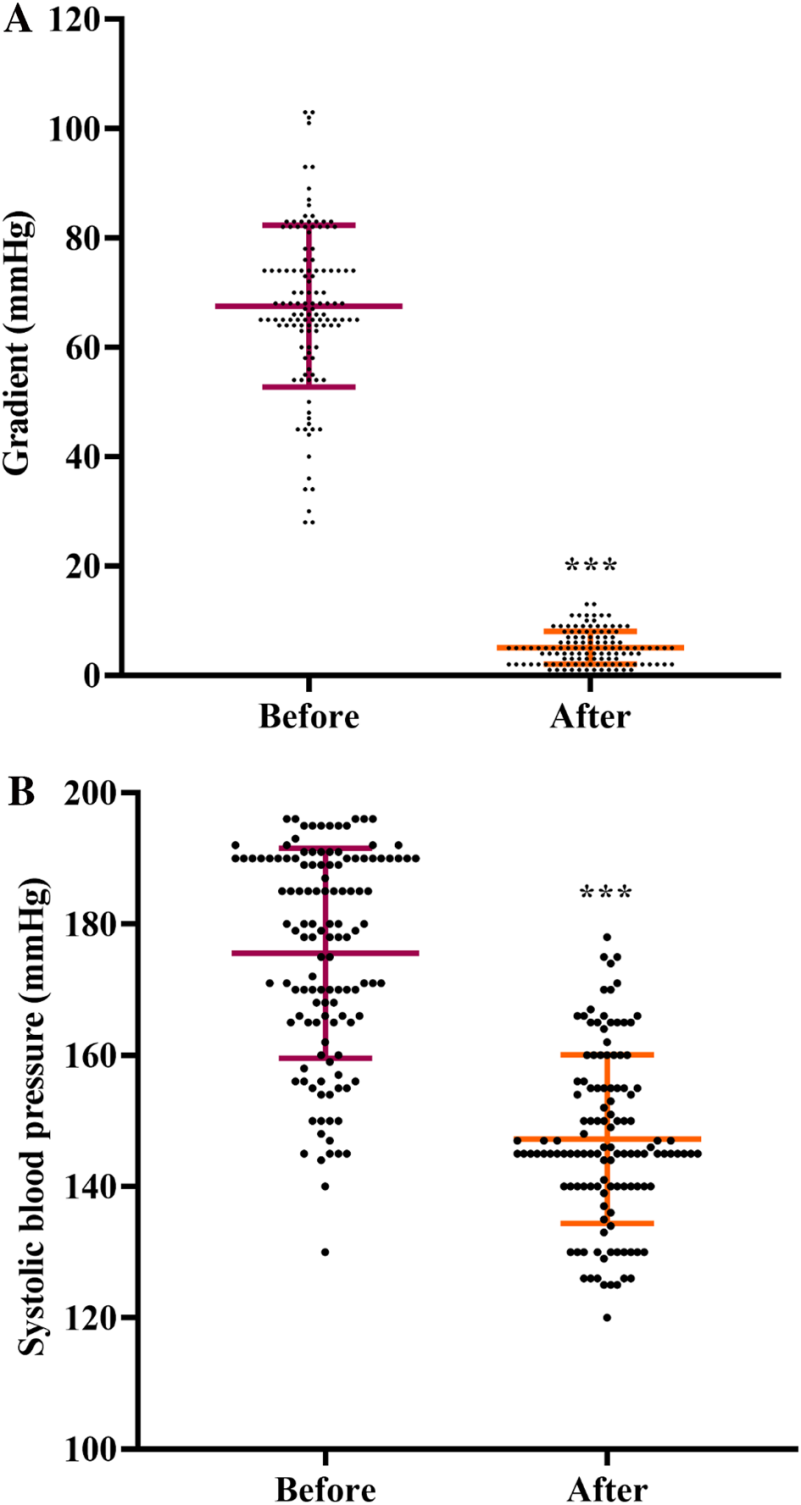
Perioperative and immediate results during self-expandable stent implantation, (**A**) gradient pressure across the coarctation site, and (**B**) systolic blood pressure. Data were presented as mean ± SD and were analyzed using paired t-test.

The gradient across the coarctation site was significantly decreased after self-expandable stenting implantation from 67.48 ± 14.79 mmHg (range 28–103 mmHg) to 5.04 ± 3.01 mmHg (range 1–13 mmHg, P < 0.001, Fig. 2A). Additionally, 80 (64%) patients had a residual gradient of less than 5 mmHg, 37 (29.6%) cases had a residual gradient between 5 and 10 mmHg, and only eight subjects (6.4%) had a residual gradient between 10 and 15 mmHg after the operation.

We also revealed that the mean SBP significantly decreased after self-expandable stenting implantation from 175.53 ± 15.99 mmHg (range 130–196 mmHg) to 147.22 ± 12.83 mmHg (range 70–178 mmHg, P < 0.001, Fig. 2B).

### The mid-term clinical outcomes

**Figure 3:**
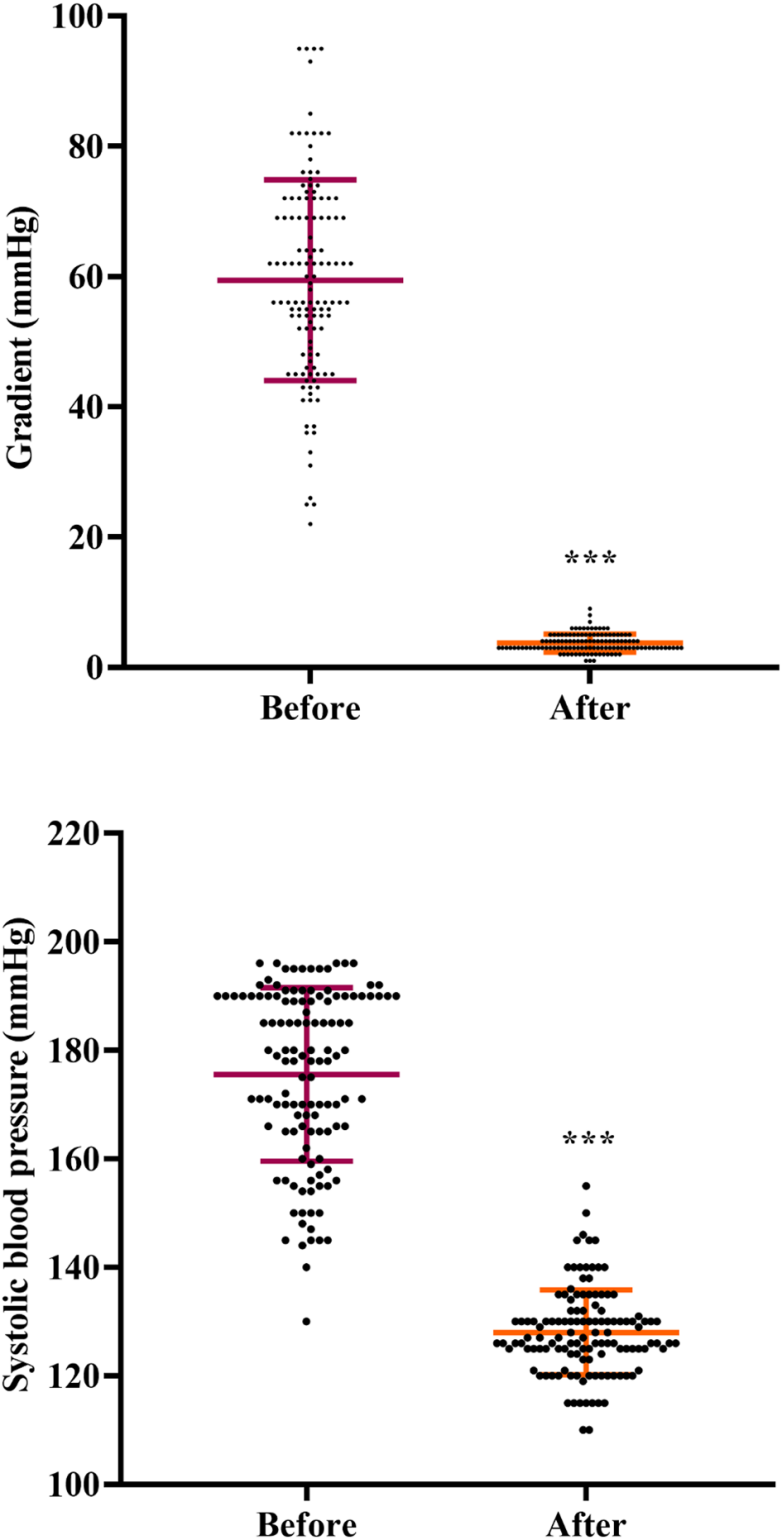
Clinical mid-term follow-up results after self-expandable stent implantation, (**A**) gradient pressure measured using echocardiography, and (**B**) systolic blood pressure. Data were presented as mean ± SD and were analyzed using paired t-test.

We also evaluated the gradient pressure after 48 ± 23.6 months (ranging from 12 to 120 months) of follow-up after stenting using echocardiography. The mean gradient was notably decreased from 59.43 ± 15.42 mmHg (range 22–95 mmHg) to 3.72 ± 1.38 mmHg (range 1–9 mmHg, P < 0.001, Fig. 3A).

In addition, the mean SBP significantly decreased over the follow-up period from 175.53 ± 15.99 mmHg (range 130–196 mmHg) to 127.99 ± 7.82 mmHg (range 110–155 mmHg, P < 0.001, Fig. 3B). Surprisingly, the therapeutic goal of SBP normalization (< 140 mmHg) was achieved in 96% of patients who underwent CoA treatment during the follow-up.

The mean number of antihypertensive drugs significantly diminished during the follow-up from 2.14 ± 0.57 (range 1–3) to 0.87 ± 0.43 (range 0–2, P < 0.001). Moreover, the antihypertensive medication dosage was reduced in all 125 patients.

### Adverse events during self-expandable stent implantation

We reported no dissection after self-expandable stent implantation in the descending thoracic aorta. However, in two patients (1.6%), we observed femoral artery dissection at the puncture site, which was immediately treated with balloon and stent implantation. Furthermore, stent dislocation during the operation was observed in two patients (1.6%), controlled by overlapping stents with a good result. These two patients were between 40 and 50 years old and had uncontrolled hypertension and severe bending in the coarctation site. One stent dislocation was due to a small stent diameter, and the other was due to a technical problem (rapid implantation).

In terms of technical or clinical complications, there were no major complications, including balloon rupture, thrombosis, aneurysmal formation, secondary stent migration, infection, death during the procedure, and in-hospital mortality.

### Mid-term adverse events

During 48 ± 23.6 months (12–120 months) of follow-up, we found no mortality, aneurysmal formation in the stent site, or dislocation. Additionally, no cases of aortic re-stenosis requiring intervention were reported according to the results of echocardiography or CT-Scan.

## Discussion

In the present study, a total of 127 self-expandable uncovered nitinol stents were successfully implanted in 125 patients with aortic Coarctation. We revealed that the gradient across the coarctation site was significantly mitigated from 67.48 ± 14.79 to 5.04 ± 3.01 mmHg after self-expandable stent implantation and to 3.72 ± 1.38 mmHg during the 48 ± 23.6 months (12–120 months) of follow-up. Similarly, Kische et al. reported a notable reduction of the pressure gradient from 54.7 ± 9.9 mmHg to 3.3 ± 2.5 mmHg following self-expandable stent implantation^22^. Zeinali et al. also noticed that the gradient across the coarctation site remarkably decreased from 62.4 ± 18 to 2.8 ± 5 mmHg after self-expandable stent implantation^15^. Another similar study showed that the peak gradient on catheterization strikingly alleviated from 59.5 ± 16.0 to 2.0 ± 5.2 mmHg after self-expandable stent implantation and to 16.9 ± 9.6 mmHg during 27.8 ± 20.9 months of follow-up^23^. Langroodi and coworkers also noticed that the gradient across the coarctation site significantly decreased from 46.26 ± 17.07 to 1.03 ± 0.19 mmHg after self-expandable stent implantation^24^. These studies’ results may align with our results regarding the amelioration of the gradient across the coarctation site in patients with aortic Coarctation treated with a self-expandable uncovered nitinol stent.

Our results showed that the SBP meaningfully attenuated from 175.53 ± 15.99 to 147.22 ± 12.83 mmHg after self-expandable stent implantation and to 127.99 ± 7.82 mmHg during mid-term follow-up. Surprisingly, 96% of patients achieved the therapeutic goal of normalizing their SBP during follow-up. Additionally, the mean number of antihypertensive drugs markedly diminished during the follow-up from 2.14 ± 0.57 (range 1–3) to 0.87 ± 0.43 (range 0–2). Moreover, the dosage of antihypertensive medications was decreased in all 125 patients. In line with our findings, Kische et al. supported that SBP significantly reduced from 162.2 ± 3.7 to 139.2 ± 12.4 mmHg after self-expandable stent implantation and to 128.1 ± 10.6 mmHg after 12 months of follow-up. Furthermore, 88.5% of patients reached the normalization of blood pressure, and the number and dosage of antihypertensive drugs significantly reduced from 2.6 to 0.9 drugs/patient during mid-term follow-up^22^.

Similarly, Zeinali et al. supported that SBP notably reduced from 166.7 ± 13 to 125.7 ± 16 mmHg during 45.5 ± 17 months of follow-up. In addition, improvement in blood pressure was observed in 84% of patients, and the antihypertensive medication dosage was attenuated in all 48 cases after self-expandable stent implantation^15^. Firoozi et al*.* also emphasized that the SBP markedly diminished from 144.2 ± 24.2 to 130.3 ± 16.3 mmHg after self-expandable stent implantation^23^. Another similar study also reported that SBP notably mitigated from 142.5 ± 17.7 to 103.1 ± 12.60 after self-expandable stent implantation^24^. The results of these studies may confirm our findings regarding improved blood pressure in patients with aortic coarctation treated with a self-expandable uncovered nitinol stent.

Neither technical nor clinical complications occurred, including balloon rupture, thrombosis, aneurysmal formation, secondary stent migration, infection, death during the procedure, and in-hospital mortality. During mid-term follow-up, we found no mortality, aneurysmal formation in the stent site, dislocation, or aortic re-stenosis requiring intervention. Similarly, Kische et al. reported no stent fracture, collapse, recoil, or secondary migration during long-term follow-up. Furthermore, one non-cardiovascular mortality was observed during 47.6 months of follow-up^22^. In the study conducted by Zeinali et al., no major complications, including aortic dissection, rupture, or vascular access difficulties, were observed following self-expandable stent implantation. During the 45.5 ± 17 months of follow-up, a total of two deaths were reported unrelated to the procedure, and there were no signs of stent fracture or aneurysm formation^15^. Moreover, Langroodi et al. observed no cases of secondary stent migration, balloon rupture, and death during the procedure, and in-hospital mortality. However, they reported one case of aneurysmal formation and three cases of aortic re-stenosis requiring intervention^24^. These studies may confirm our results regarding the safety of treatment of aortic Coarctation using a self-expandable uncovered nitinol stent.

Stent primary migration during the operation was observed in two patients (1.6%), controlled by overlapping stents with a good result. Similar articles also reported a few numbers of stent dislocation by self-expanding uncovered nitinol stents. This may be due to the anti-jump specification of the self-expanding stents in order to prevent sudden stent jumps and retain stability^25,26^. Furthermore, the self-expanding uncovered nitinol stents have a maximal radial force, leading to adequate flexibility. In addition, radiopaque markers are used on the inner sheath to facilitate the placement of the stent^27^. Interestingly, self-expanding stents are considered smart stents that cause a progressive drop in the gradient over time after the stent implantation^28^.

Haddad et al. investigated the results of bare-metal and polytetrafluoroethylene (PTFE)-covered Optimus stents in congenital heart disease (CHD) interventions. Optimus is a balloon-expandable, hybrid cell-designed, cobalt–chromium vascular stent. They evaluated 170 patients with CHD consisting of right ventricular outflow tract (RVOT) stenting (106 patients), aortic coarctation (26 patients), fontan-circulation fenestration closure (21 patients), and miscellaneous (17 patients). They concluded that Optimus stents are safe, effective, and reliable tools with promising short-to-midterm outcomes in treating simple and complex CHDs by transcatheter^29^. Another study also supported the idea that aortic coarctation can be treated safely and effectively with stent placement. They also reported that a favorable outcome had been associated with Cheatham Platinum stents^30^.

Different studies have shown that balloon expandable stents are preferable to surgery or balloon dilation. There is also a risk of complications, including aortic rupture or dissection, stent migration, balloon rupture, or complications related to vascular access^12,31^. Besides that, self-expanding stents have also been used because they are flexible, adaptable to aortic anatomy, and easy to deploy. Moreover, due to their low constant radial force, these stents may cause stenoses to gradually widen as well as cause aortic tissue to be less injured^22,32^. In both early and long-term follow-ups, nitinol self-expandable stents have proven safe and effective for treating aortic coarctation and, as such, may be accepted for long-term management as an effective alternative to surgery, balloon angioplasty, and balloon-expandable stents. The aortic wall apposition is better with self-expandable stents; it is easier to advance them across coarctations, and there are fewer aortic wall complications^15,31^.

## Conclusion

Treatment of aortic Coarctation using a self-expandable uncovered nitinol stent is safe and effective during the perioperative to mid-term follow-up. Its peculiar design maintains adequate localized radial strength over time, minimizes trauma to the adjacent aortic wall, and results in minimal device-related complications. Furthermore, blood pressure control is optimized immediately and persistently even after mid-term follow-up. Therefore, a self-expandable nitinol stent may be an excellent alternative method to surgical procedures, balloon angioplasty, or balloon-expandable stents in terms of mid-term management.

## Data Availability

Upon a reasonable request, the corresponding author will provide the data supporting the findings of this study**.**
